# Signal Denoising Method Based on EEMD and SSA Processing for MEMS Vector Hydrophones

**DOI:** 10.3390/mi15101183

**Published:** 2024-09-24

**Authors:** Peng Wang, Jie Dong, Lifu Wang, Shuhui Qiao

**Affiliations:** School of Mathematics, North University of China, Taiyuan 030051, China; s202208023@st.nuc.edu.cn (J.D.); s202208038@st.nuc.edu.cn (L.W.); s202208021@st.nuc.edu.cn (S.Q.)

**Keywords:** ensemble empirical mode decomposition (EEMD), singular spectrum analysis (SSA), micro-electronic mechanical systems (MEMS) vector hydrophone

## Abstract

The vector hydrophone is playing a more and more prominent role in underwater acoustic engineering, and it is a research hotspot in many countries; however, it also has some shortcomings. For the mixed problem involving received signals in micro-electromechanical system (MEMS) vector hydrophones in the presence of a large amount of external environment noise, noise and drift inevitably occur. The distortion phenomenon makes further signal detection and recognition difficult. In this study, a new method for denoising MEMS vector hydrophones by combining ensemble empirical mode decomposition (EEMD) and singular spectrum analysis (SSA) is proposed to improve the utilization of received signals. First, the main frequency of the noise signal is transformed using a Fourier transform. Then, the noise signal is decomposed by EEMD to obtain the intrinsic mode function (IMF) component. The frequency of each IMF component in the center further determines that the IMF component belongs to the noise IMF component, invalid IMF component, or pure IMF component. Then, there are pure IMF reserved components, removing noisy IMF components and invalid IMF components. Finally, the desalinated IMF reconstructs the signal through SSA to obtain the denoised signal, which realizes the denoising processing of the signal, extracting the useful signal and removing the drift. The role of SSA is to effectively separate the trend noise and the periodic vibration noise. Compared to EEMD and SSA separately, the proposed EEMD-SSA algorithm has a better denoising effect and can achieve the removal of drift. Following that, EEMD-SSA is used to process the data measured by Fenhe. The experiment is carried out by the North University of China. The simulation and lake test results show that the proposed EEMD-SSA has certain practical research value.

## 1. Introduction

The hydrophone is widely used in ocean exploration as a type of transducer for measuring and receiving signals in water. Hydrophones can be divided into subtypes such as undirected, directional, sound pressure, vibration speed, electromotive, piezoelectric, magnetostrictive, and so on, according to the different working principles, energy exchange principles, characteristics, and structures used. At present, the acoustic pressure scalar hydrophone and the acoustic pressure hydrophone array are mainly used in underwater exploration engineering. As the in-depth study of the ocean has continued, many deficiencies have been gradually shown in the acoustic pressure hydrophone and its array. For example, when detecting targets with a low signal-to-noise ratio, the most commonly used method is to use a large-scale hydrophone array to measure the sound pressure at multiple points in the sound field and then filter the values in the spatial domain to improve the spatial gain so as to obtain a better detection performance. However, as the frequency of the acoustic signal decreases, the aperture of the array will gradually increase under the condition of maintaining a certain gain and beamwidth, which affects the engineering practicality of the underwater acoustic system to a certain extent.

The vector hydrophone is developed on the basis of the scalar hydrophone, which is a new sensor for detecting the direction and intensity of acoustic energy flow underwater [1,2]. The micro-electronic mechanical systems (MEMS) vector hydrophone is a novel representative biomimetic sensor [3,4,5] based on the meso-piezoresistive effect and the acoustic theory of cylinders. In energy detection, the system used by a vector hydrophone has a stronger ability to resist same-sex noise, so it can realize multi-target recognition in far-field applications. The role of the vector hydrophone in underwater acoustic engineering has become more and more prominent, and its signal processing technology is one of the most remarkable research directions in the field of underwater acoustic engineering [6,7].

In order to obtain the sound source information better, the signal-to-noise separation of the vector hydrophone signal is needed. Methods for effectively removing these noises and interferences are also an important research topic in underwater acoustic signal processing. There are quite a number of studies on underwater acoustic signal denoising [8], wavelet packet denoising [9], adaptive filtering [10], morphological filtering [11], empirical mode decomposition (EMD) [12], etc. These methods have their own advantages in terms of denoising, but there are also some shortcomings [13].

In order to remove all types of noise in the signal, many methods are used. Based on classical finite impulse response (FIR) [14] and infinite impulse response (IIR) [15], a denoising method is applied to non-stationary signals in time, but it does not always perform well. The Kalman filtering method is widely used to denoise the random drift signal of micro-mechanical systems. However, the Kalman filtering method may have the phenomenon of filter divergence due to the inaccuracy of the error model [16]. In [17], an empirical mode decomposition (EMD) method was proposed based on the work of Mr. Huang E in 1998 [18]. It has been widely studied in the field of filtering and denoising [19,20]. As a signal processing method that can adaptively decompose nonlinear and non-stationary signals, it decomposes one-dimensional or two-dimensional signals into a series of intrinsic mode functions [21,22]. However, the EMD method lacks strict mathematical proof, and faces theoretical challenges such as serious mode aliasing and endpoint effect problems, and the options for interpolation methods and stop criteria are not unique [23]. In [24], Wu et al. proposed an integrated empirical ensemble empirical mode decomposition (EEMD) method by adding different Gaussian white noise to the original signal. This is a noise-assisted signal processing method that can effectively attenuate the modal aliasing phenomenon that exists in the EMD method. This method mainly uses the statistical characteristics of zero-mean white noise and the uniform frequency distribution. In the process of signal decomposition, Gaussian white noise is added as the background [25]. Through multiple iterations, signals of different time characteristic scales are automatically mapped to the scales related to white noise [26]. In this way, the anti-mixing effect is improved. In the process of multiple iterations, white noise can be added for noise cancellation so as to approach the real modes and achieve an anti-aliasing effect [27]. The complementary ensemble empirical mode decomposition (CEEMD) adds pairs of white noises that are opposite to each other to optimize noise processing [28]. The only difference between EEMD and CEEMD lies in the method for adding white noise. EEMD adds independent white noise, which may not completely eliminate the influence of noise, but its independence and randomness help reduce the interference of noise in the averaging process. Therefore, the independent noise addition method and intuitiveness of EEMD are more flexible than CEEMD. And, when dealing with shorter or simpler signals, EEMD has a higher computational efficiency than CEEMD. The variational mode decomposition (VMD) method was proposed by Dragomiretskiy, K in 2014. It decomposes signals by solving variational problems through optimization algorithms, but its implementation process is relatively complex [29].

Singular spectrum analysis (SSA) is an algorithm that separates the feature quantity in the noise signal from the noise and uses the feature quantity to reconstruct the signal to achieve noise reduction, effectively separating the trend and periodic vibration noise [30]. Through the use of SSA, operations like eliminating noise by reconfiguring a new signal using the appropriate isolated modes can be performed. The calculation of SSA is not suitable for basic functions, unlike Fourier series expansion or wavelet series expansion, and it does not require an assumption of a definite model, as AR algorithms do. Therefore, SSA allows us to analyze the structural changes of a signal [31]. In [32], zero mean signals were normalized to the unit energy signals, then singular spectrum analysis was performed and the sets of singular spectrum analysis components were obtained. In [33], the authors extracted the blood volume pulse signal to raw facial RGB trace signals, then the spectra for the interference of illumination variations were extracted from the raw signal obtained from facial regions of interest using singular spectrum analysis. In [34], SSA was based on a nonparametric time series analysis technique and decomposed a signal into a number of reconstructive components (RCs) from which data trends and oscillatory components may be extracted. SSA can not only be used in ECG signal denoising, but also in fault diagnosis. In this study, a new method based on singular spectrum analysis and EMD is proposed for fault detection in chemical process systems, where SSA extracts the trends of process signals using the eigenvalues of trajectory matrices. In [35], the SSA technique was adopted to decompose and reconstruct water consumption in relation to six weather variables to create a seasonal and stochastic time series; this climate factor assessment provided a new method for quantifying water demand.

Therefore, a new method for denoising MEMS vector hydrophones using EEMD and SSA is proposed in this paper. Based on the reconstructed signal of EEMD, SSA is carried out to achieve the purpose of removing residual noise.

The rest of the article is organized as follows: in the Section 2, the theoretical basis and specific steps of EEMD, the SSA decomposition method, and the proposed algorithm are introduced. In the Section 3, simulations of the proposed algorithm are conducted and compared with other algorithms. The application of the proposed algorithm to MEMS hydrophones and the conclusions are in Section 4 and Section 5, respectively.

## 2. Methodology

### 2.1. Ensemble Empirical Mode Decomposition

The main idea of the EEMD method is that the average value of a certain component measured many times in statistics can improve the accuracy of measurement. EEMD decomposes a set of intrinsic mode function (IMF) components by adding enough groups of different white noise to the original signal several times and then uses the random characteristics of white noise with a mean value of zero to calculate the overall average of each IMF component obtained by EMD as an EEMD IMF component so as to eliminate the influence of white noise. White noise can provide a relatively consistent reference scale distribution for EMD and ensure the continuity of each mode function in the time domain to reduce modal aliasing, which is considered to be an important achievement in EMD improvement. The specific decomposition steps of EEMD are as follows [24].

(1) A group of random white noise ω(t) sequences with zero mean value and equal variance are added to the original signal M(t), and we obtain a new signal

(1) M(t)=s(t)+ω(t).

(2) The signal M(t) is decomposed using EMD into a series of IMF components and residual components $r_{n}\left(t\right)$, and then

(2) $$M\left(t\right)=\sum_{j=1}^{N}\mathrm{imf}\left(j\right)+r_{n}\left(t\right).$$

(3) Add different white noise each time, repeat the above steps, and obtain

(3) $$M_{i}\left(t\right)=\sum_{j=1}^{N}\mathrm{imf}\left(ij\right)+r_{m}\left(t\right).$$

(4) In order to eliminate the influence caused by the repeated addition of Gaussian white noise, the actual IMF component was obtained through the overall average calculation of the above results

(4) $${\mathrm{imf}}_{j}=\frac{1}{n}\sum_{i=1}^{n}{\mathrm{imf}}_{ij}.$$

### 2.2. Singular Spectrum Analysis

Singular spectrum analysis (SSA) is a model-free and data-driven time series decomposition method, which decomposes a time series into three components: trend, seasonal components, and noise. These components are generated based on the covariance property of the original time series data, and they are independent and additive in nature. SSA consists of two complementary stages: decomposition and reconstruction.

(1) Decomposition: Select a window length *L* (not greater than $\frac{N}{2}$), and convert the one-dimensional time series $Y=(y_{0},\cdots ,y_{N-1})$ into a multi-dimensional trajectory matrix of L×K:

(5) $$X=\left[\begin{array}{cccc} y_{0} & y_{1} & \cdots & y_{k-1}\\ y_{1} & y_{2} & \cdots & y_{K}\\ \vdots & \vdots & \ddots & \vdots \\ y_{L-1} & y_{L} & \cdots & y_{N-1} \end{array}\right],$$

where K=N−L+1. Calculate $XX^{T}$, perform singular value decomposition (SVD) on it, and obtain its *L* eigenvalues $\lambda_{1}\geq \lambda_{2}\cdots \geq \lambda_{L}\geq 0$ and the corresponding eigenvectors $u_{1},u_{2},\cdots ,u_{L}$; then, $X=X_{1}+X_{2}+\cdots +X_{d}$, where $X_{i}=\sqrt{\lambda_{i}u_{i}V_{i}^{T}}$ represents the *i*-th SVD component, $V_{i}=X^{T}u_{i}/\sqrt{\lambda_{i}}$, $d=\max\left\{j:\lambda_{i}>0\right\}$, and each eigenvalue represents the energy contribution of the corresponding SVD component to the original signal [36] so the energy contribution of the SVD component to the original signal decreases as the order increases.

(2) Reconstruction: First, select a certain number of SVD components to form a subset $X_{I}$, I∈{1,2,⋯,d}, as needed. $X_{I}=\sum_{j\in I}\sqrt{\lambda_{j}u_{j}V_{j}^{T}}$ and $\sum_{j\in I}\lambda_{j}/\sum_{i=1}^{d}\lambda_{i}$ is called the contribution rate of $X_{I}$; then, the matrix $X_{I}$ of L×K is reconstructed into a time series $G=(g_{0},\cdots ,g_{N-1})$ of length *N* and the time series *G* is the target signal extracted from the time series *Y*, which may represent the trend, noise, and denoising signal in *Y*. The specific way of reconstructing the time series *G* from the matrix $X_{I}$ is as follows: $L^{*}=\min\left\{L,K\right\}$, if K≥L, then $X_{I}^{*}=X_{I}$, and if L>K, then $X_{I}^{*}=X_{I}^{T}$. $X_{m,n}$ represents the element of the *m*-th row and *n*-th column in the matrix $X_{I}^{*}$, and each data point in the time series *G* can be obtained using the following formula:

(6) $$g_{n}=\left\{\begin{array}{ccc} \frac{1}{n+1}\sum_{m=1}^{n+1}X_{m,n-m+2}, & \; & 0\leq n\leq L^{*}-1,\\ \frac{1}{L^{*}}\sum_{m=1}^{L^{*}}X_{m,n-m+2}, & \; & L^{*}-1\leq n\leq K^{*},\\ \frac{1}{N-n}\sum_{m=n-K^{*}+2}^{N-K^{*}+1}X_{m,n-m+2}, & \; & K^{*}\leq n<N. \end{array}\right.$$

Since the ambient noise is a random signal that changes less in all directions, the eigenvectors corresponding to the smaller singular values are caused by environmental noise, and these singular values and their corresponding eigenvectors can be removed to eliminate the environmental noise. The selection criteria of eigenvalues meet the following formula

(7) $$\frac{\frac{\lambda_{r-1}-\lambda r}{r-(r-1)}}{\frac{\lambda_{r}-\lambda_{d}}{d-r}}>1,$$

and $\lambda_{r}\leq 0.05$, r<d, where $d=\max\left\{j:\lambda_{j}>0\right\}$. $\lambda_{r}$ and $\lambda_{d}$ represent the *r*-th and *d*-th singular values in descending order, respectively. $\frac{\lambda_{r-1}-\lambda r}{r-(r-1)}$ represents the rate of change of $\lambda_{r-1}$ and $\frac{\lambda_{r}-\lambda_{d}}{d-r}$ represents the rate of change from $\lambda_{r}$ to $\lambda_{d}$. Find the point where the singular value tends to be flat and the singular value of the point is less than 0.05, that is, find the value of *r* first. If the slope ratio is greater than 1, the change rate of λr is less than $\lambda_{r-1}$. The singular value with a subscript greater than *r* is removed, that is, the singular value with value less than λr and its corresponding eigenvector are removed.

### 2.3. Ensemble Empirical Mode Decomposition—Singular Spectrum Analysis

The modes obtained through EEMD possess a series of unique properties, specifically including adaptability, nonlinearity, clear physical meaning, orthogonality, and completeness. These properties endow EEMD with certain advantages in processing complex signals such as underwater acoustic signals. Nevertheless, for broadband signals, EEMD’s noise suppression capability may be relatively weaker. This is because broadband signals typically contain multiple frequency components with indistinct boundaries between them. However, the noise suppression ability can be improved by combining EEMD with other methods. SSA, on the other hand, excels in noise suppression and feature extraction, effectively eliminating noise interference from signals. Therefore, the EEMD-SSA algorithm can fully leverage the advantages of both methods to enhance the effectiveness and precision of signal denoising while also improving the accuracy and reliability of signal analysis. This combined approach holds broad application prospects in processing complex, nonlinear, and non-stationary underwater acoustic signals.

**Figure 1:**
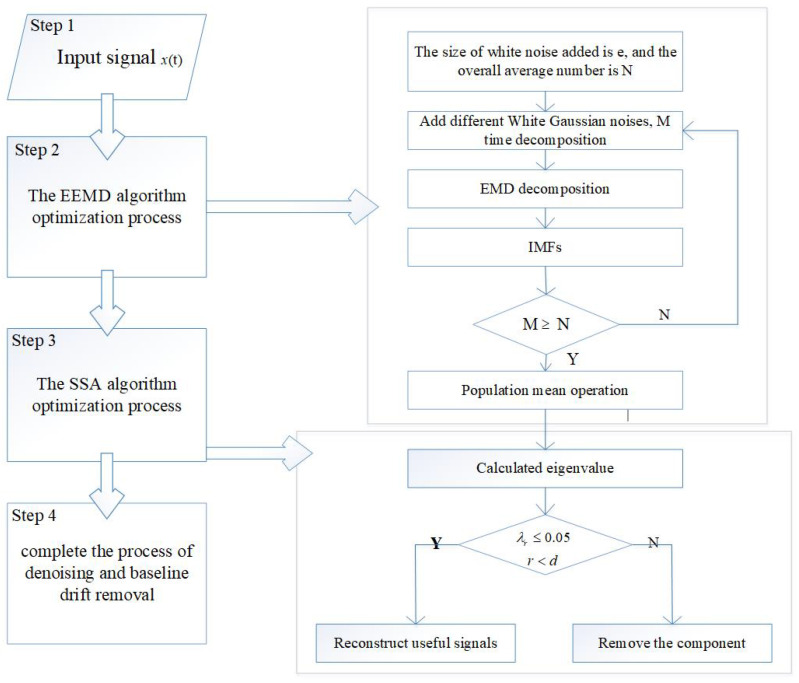
Flowchart of the EEMD-SSA algorithm.

In conclusion, based on the above analysis and theoretical basis, the EEMD-SSA algorithm is proposed for noise reduction in this paper. The noise signal is decomposed using EEMD to obtain the modal components. The components similar to the original signal spectrum are selected for reconstruction, and then the noise reduction is processed by SSA. The EEMD-SSA algorithm proposed in this paper can be divided into four steps, and the flow chart of the EEMD-SSA algorithm can be seen in Figure 1.

(1) Input the original signal.

(2) Gaussian white noise is added for EMD to obtain a series of IMF components, and then the population average is calculated to eliminate the effect of multiple white noise additions.

(3) The reconstructed signal is filtered using the SSA algorithm. The above IMF matrix is used to calculate the covariance matrix of component composition and obtain a more singular value and its corresponding eigenvectors. This is due to environmental noise; when there are small changes to random signals in all directions, the characteristics corresponding to the smaller singular value are caused by environmental noise. So, the singular value vector and its characteristics are removed to achieve denoising.

(4) The result of denoising is obtained.

## 3. Simulation

In order to verify the effectiveness and advantages of the proposed algorithm in underwater acoustic signal denoising, through analysis of the characteristics of underwater acoustic signal noise, this section will carry out simulation experiments on the algorithm proposed in this paper through analog signal.

### 3.1. Simulation Experiment 1

**Figure 2:**
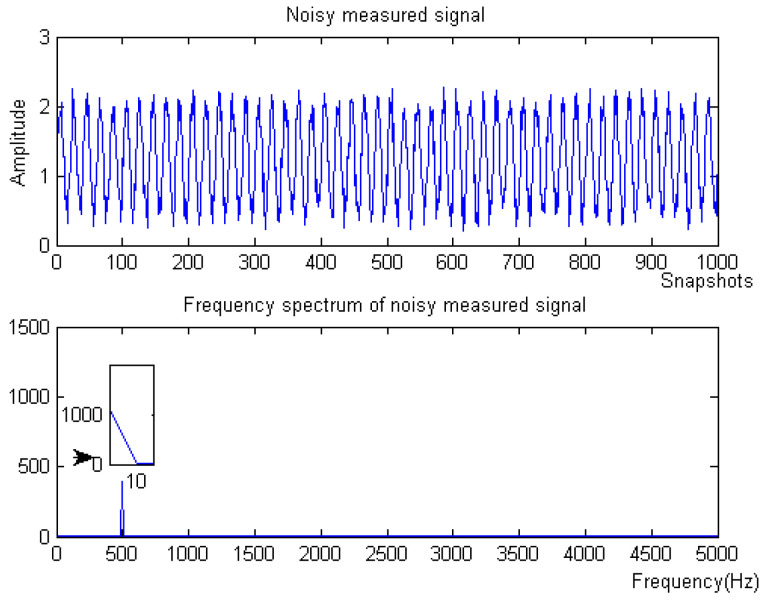
Original signal of Equation (8).

In this section, the simulation signal *S* consists of the target signal *f*, other frequency signals $f_{1},\;f_{2},\;f_{3}$, the baseline drift signal $f_{4}$, and 0.5 times the standard Gaussian white noise *n*, as shown in the following equation:

(8) $$\left\{\begin{array}{c} f=2\sin(2\pi \cdot 500t),\\ f_{1}=0.8\sin\left(2\pi \cdot 5t\right),\\ f_{2}=0.6\sin\left(2\pi \cdot 100t\right),\\ f_{3}=0.3\sin\left(2\pi \cdot 2000t\right),\\ f_{4}=1,\\ n=0.5rand(t),\\ S=f+f_{1}+f_{2}+f_{3}+f_{4}+n, \end{array}\right.$$

where *f* is the target signal with a frequency of 500 Hz, and $f_{1},\;f_{2},\;f_{3}$ are the signals with frequencies of 5 Hz, 100 Hz, and 2 kHz, respectively. $f_{4}$ is the baseline drift signal and the sampling frequency is 10 kHz. The simulation signal contains obvious noise and baseline drift. The waveform of the measured noise signal and the spectrum of the measured noise signal are shown in Figure 2.

**Figure 3:**
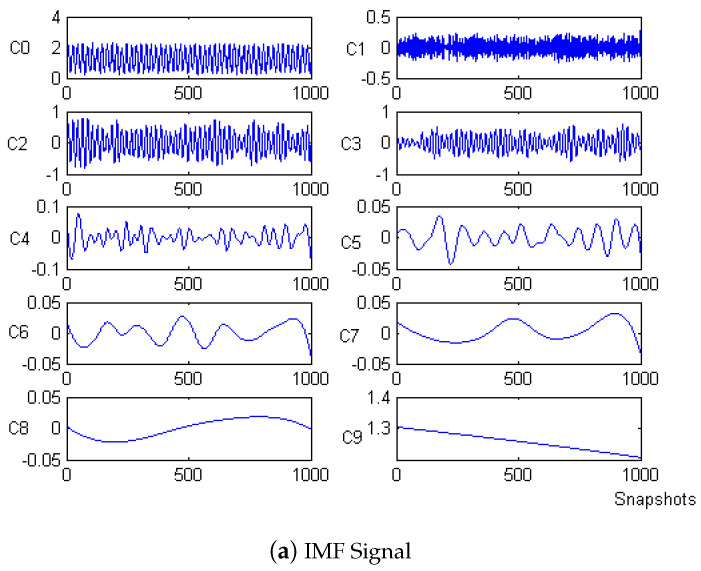

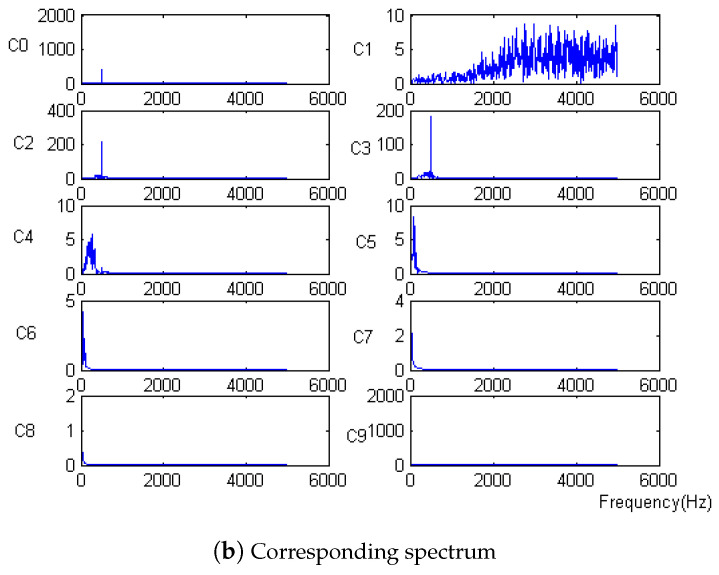
IMF signal and corresponding spectrum of Equation (8).

**Figure 4:**
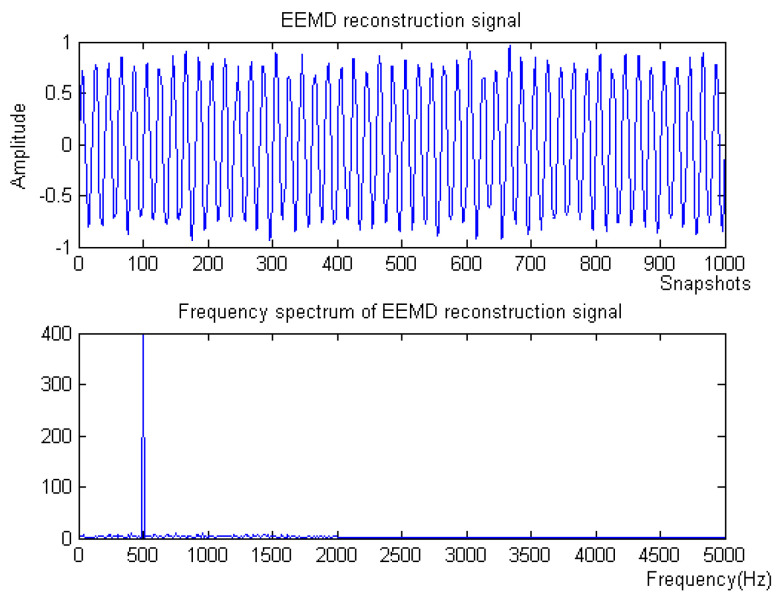
The denoising result of EEMD algorithm of Equation (8).

Firstly, the measured noise signal is decomposed by EEM. The time–frequency diagram is shown in Figure 3. $C_{0}$ is the original signal, $C_{1}$–$C_{9}$ is ${\mathrm{IMF}}_{1}$–${\mathrm{IMF}}_{9}$, $C_{1}$ and $C_{2}$ have the lowest energy, and the spectrum after $C_{5}$ is unstable. Therefore, $C_{3}$ and $C_{4}$ are selected to be reconstructed, and the spectrum of reconstructed signal is shown in Figure 4.

**Figure 5:**
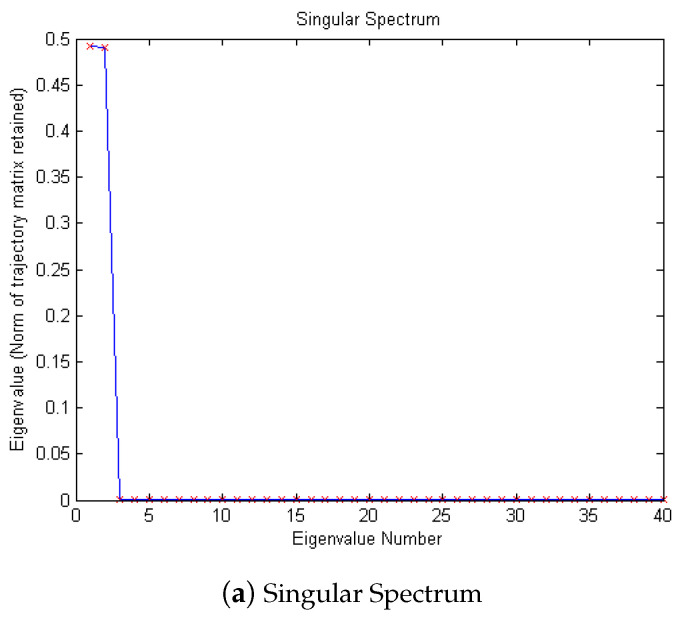

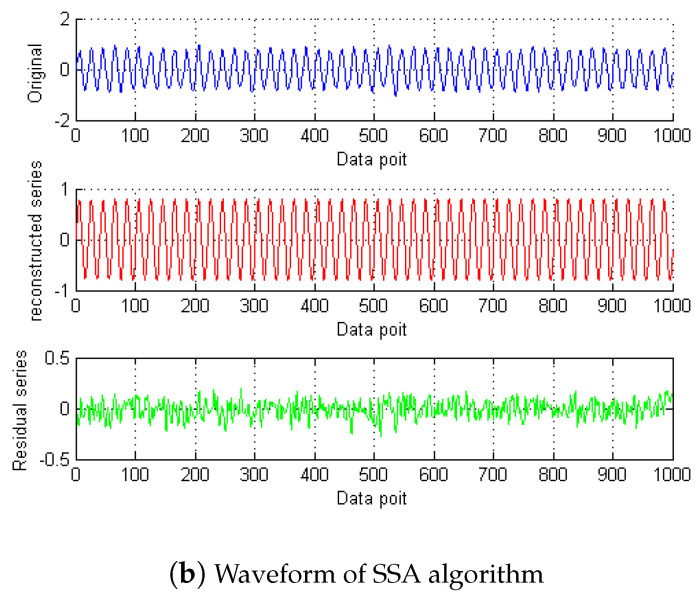
The denoising result of SSA algorithm of Equation (8).

Then, SSA is used for noise reduction to obtain the eigenvalue of SSA and the final filtered signal and spectrum, as shown in Figure 5.

**Figure 6:**
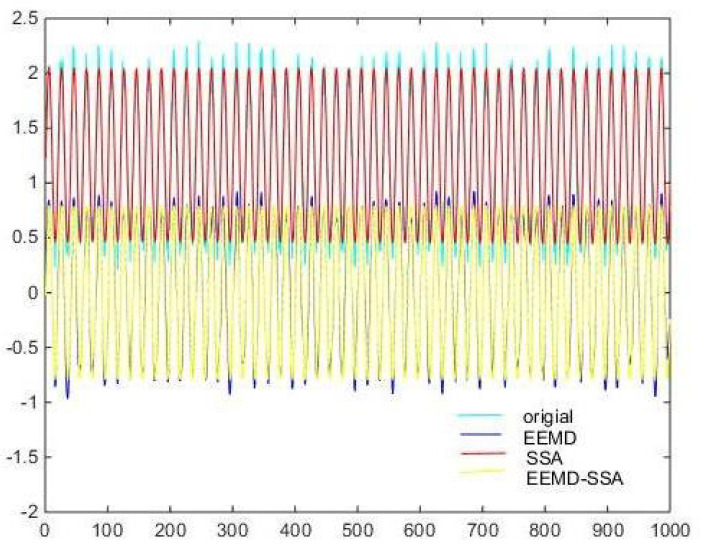
Time domain signals of different methods of Equation (8).

**Figure 7:**
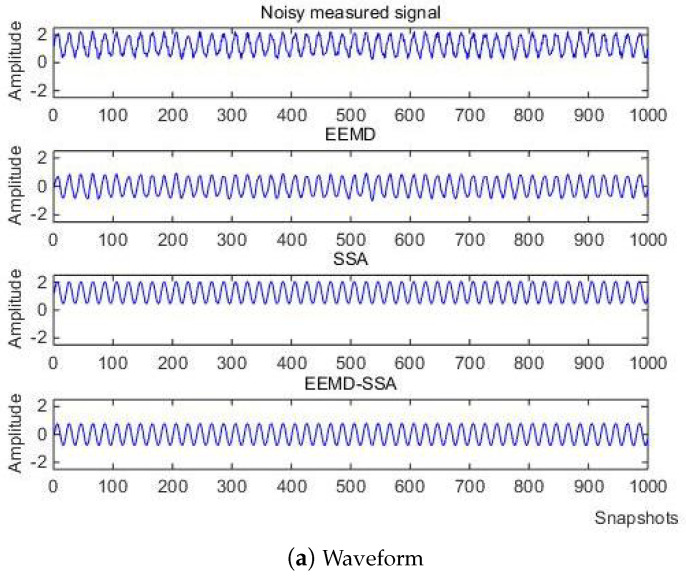

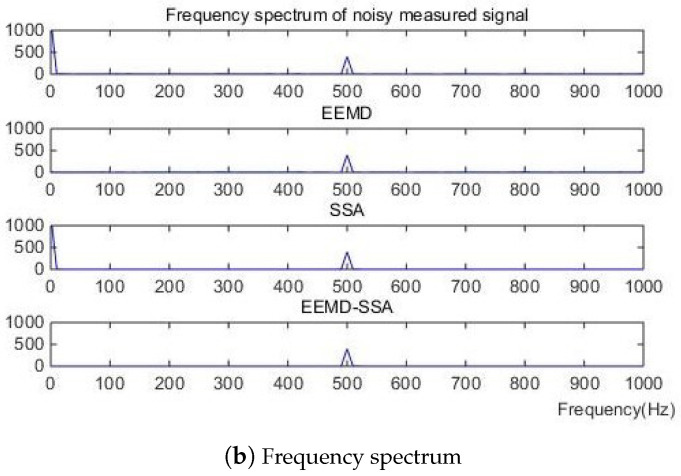
Comparison of denoising results of different algorithms of Equation (8).

According to the eigenvalue, the first singular is 0.4913, the second is 0.4894, and the third is 8.5502 × $10^{-4}$. So, according to principal component analysis, the eigenvalue after the second one is too small to be ignored. After removing these singular values, the denoising effect is realized. In Figure 6 and Figure 7, it can be seen from the time domain and frequency domain graphs that the filtering effect is obvious.

### 3.2. Simulation Experiment 2

Due to the influence of the lake environment and human activities, the noise intensity of the underwater acoustic signal is variable. To simulate this situation, the simulated signal *S* consists of the target signal *f* and the baseline drift signal $f_{1}$ and Gaussian white noise at different decibel levels. The specific form is as follows:

(9) $$\left\{\begin{array}{c} f=2\cos(2\pi \cdot 100t)(\sin(2\pi \cdot 5t)+2),\\ f_{1}=3,\\ S=f+f_{1}+n, \end{array}\right.$$

Here, the sampling frequency is 10 kHz, the target signal *f* is composed of an amplitude modulation signal, the noise signal *n* is white noise at different decibel levels, and the noise signals are Gaussian white noises of −10 dB and 10 dB for which the calculation formula for signal-to-noise ratio (SNR) is shown in Equation (10).

(10) $$\;SNR=10\cdot \mathrm{lg}\frac{P_{s}}{P_{n}},$$

where $P_{s}$ represents the power of signal and $P_{n}$ denotes the power of noise.

**Figure 8:**
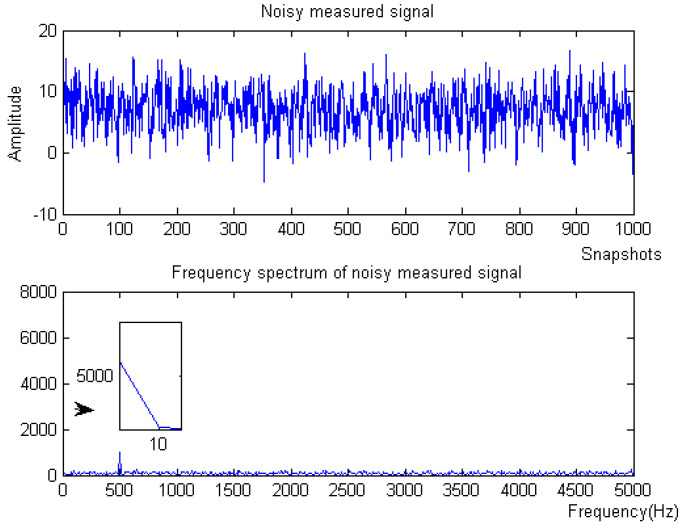
Original signal of Equation (9) with −10 dB.

(1) When the noise signal is −10 dB, the waveform and spectrum of the simulation signal are shown in Figure 8.

**Figure 9:**
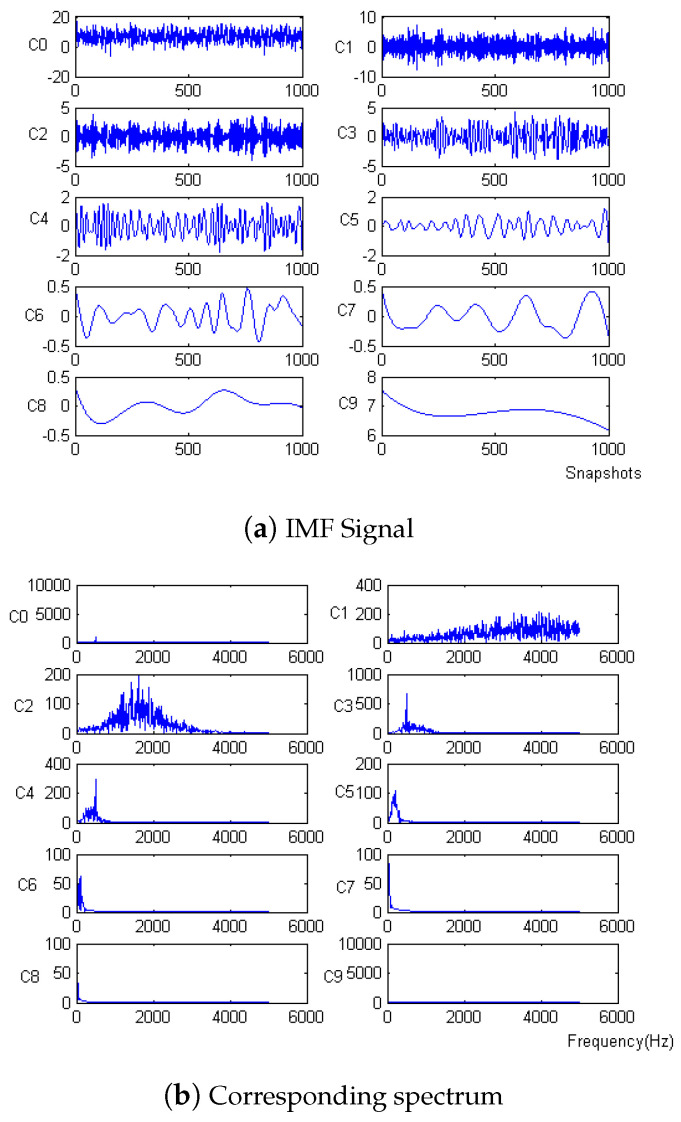
IMF signal and corresponding spectrum of Equation (9) with −10 dB.

**Figure 10:**
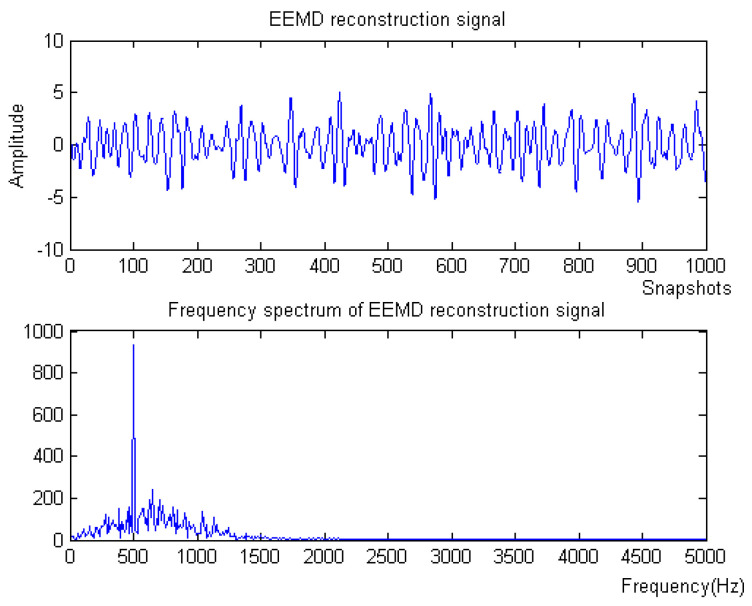
The denoising result of EEMD algorithm of Equation (9) with −10 dB.

Firstly, the measured noise signal is decomposed using EEMD, and the time–frequency diagram is shown in Figure 9. In Figure 9, $C_{0}$ is the original signal, $C_{1}$–$C_{9}$ is ${\mathrm{IMF}}_{1}$–${\mathrm{IMF}}_{9}$, $C_{1}$, $C_{2}$ and $C_{3}$ have the lowest energy, and the frequency after $C_{6}$ is very small compared to the original signal and can be ignored, which has no effect on the result. Therefore, $C_{4}$ and $C_{5}$ are selected to be reconstructed, and the spectrum of reconstructed signal is shown in Figure 10.

**Figure 11:**
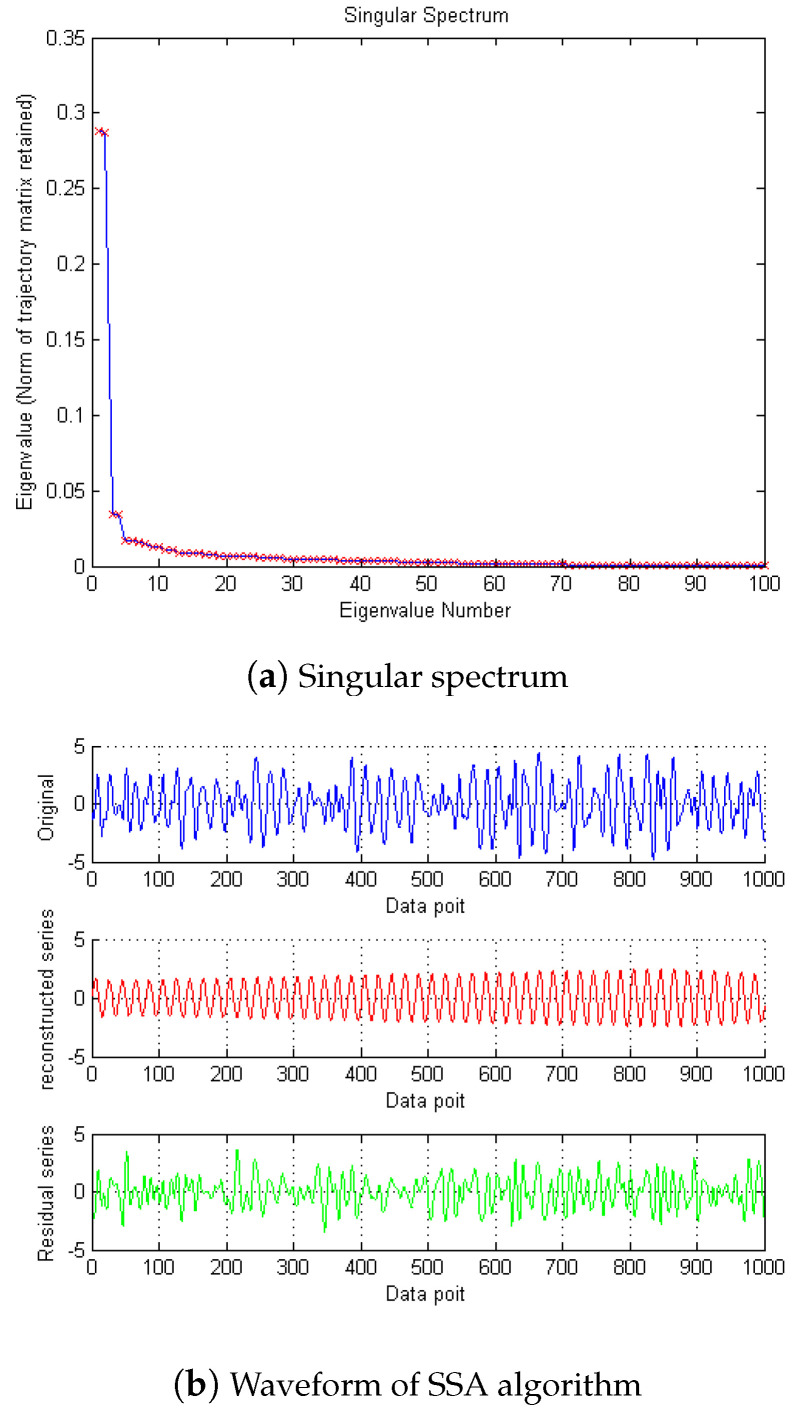
The denoising result of SSA algorithm of Equation (9) with −10 dB.

Then, SSA is used for noise reduction to obtain the characteristic values of SSA and the final filtered signal and spectrum, as shown in Figure 11.

**Figure 12:**
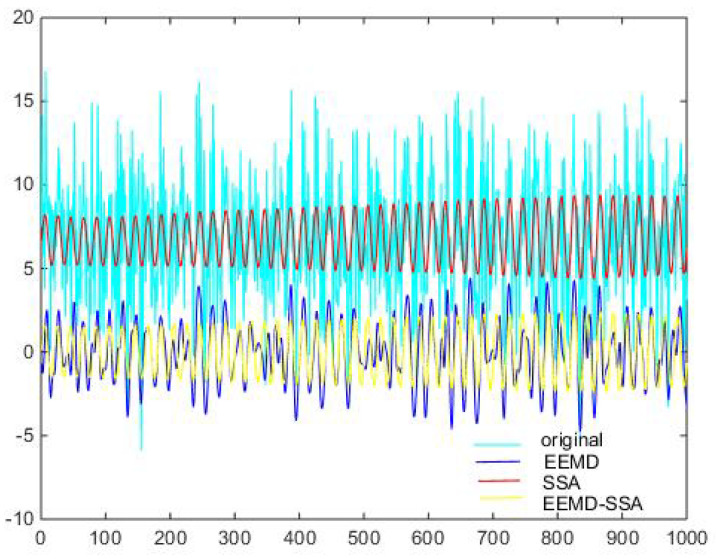
Time-domain signals of different methods of Equation (9) with −10 dB.

**Figure 13:**
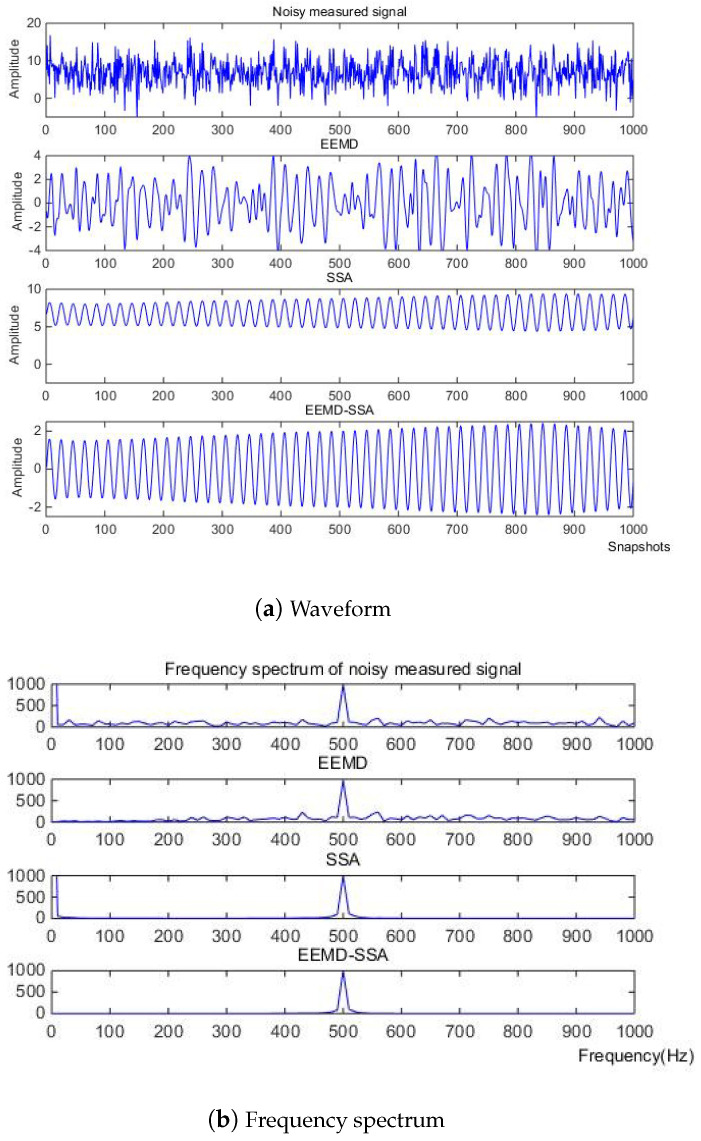
Comparison of denoising results of different algorithms of Equation (9) with −10 dB.

According to the eigenvalue, the first singular is 0.2556, the second is 0.2542, and the third is 0.0310. The eigenvalue after the second one is too small to be ignored. After removing these singular values, the denoising effect of the MEMS hydrophone is realized. In Figure 12 and Figure 13, it can be seen from the time domain and frequency domain graphs that the filtering effect is obvious.

Denoising and baseline drift correction analysis: There is a lot of burring noise and significant baseline drift in the original signal in Figure 8. As can be seen from Figure 10, the baseline drift is removed in the spectrum diagram reconstructed by the EEMD algorithm, but there is still a lot of burring noise in the upper and lower parts of the waveform. In Figure 10, the signal waveform processed by the SSA algorithm has obvious baseline drift in the upper position, but the burring noise is significantly improved. Therefore, the algorithm proposed in this paper first removes baseline drift using EEMD and then denoises the signal; then, the waveform is in the middle position, which is closer to the target signal.

**Figure 14:**
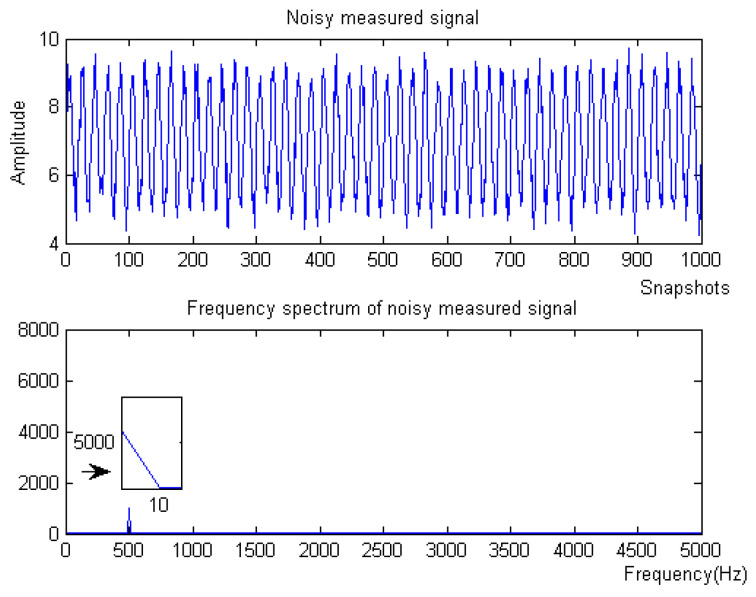
Original signal of Equation (9) with 10 dB.

(2) When the noise signal is 10 dB, the waveform, spectrum of the simulation signal are shown in Figure 14.

**Figure 15:**
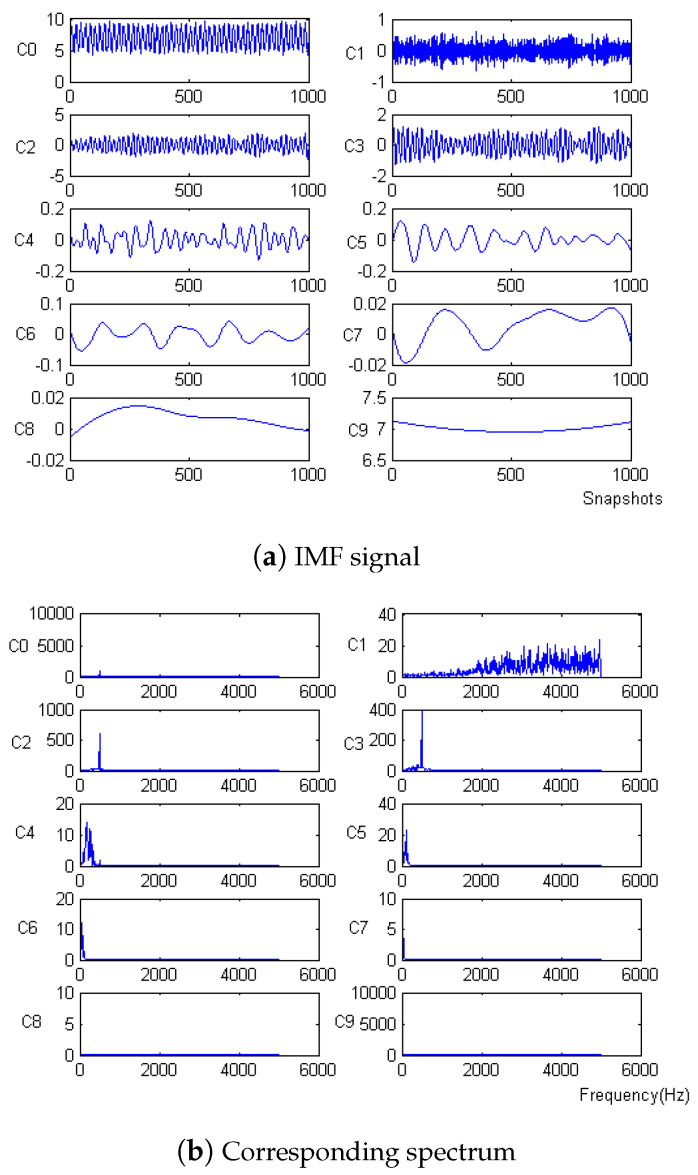
IMF signal and corresponding spectrum of Equation (9) with 10 dB.

**Figure 16:**
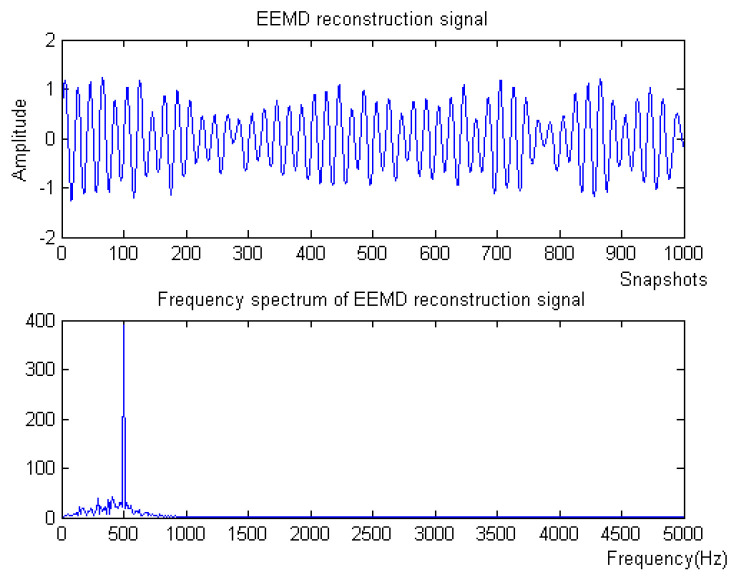
The denoising result of EEMD algorithm of Equation (9) with 10 dB.

**Figure 17:**
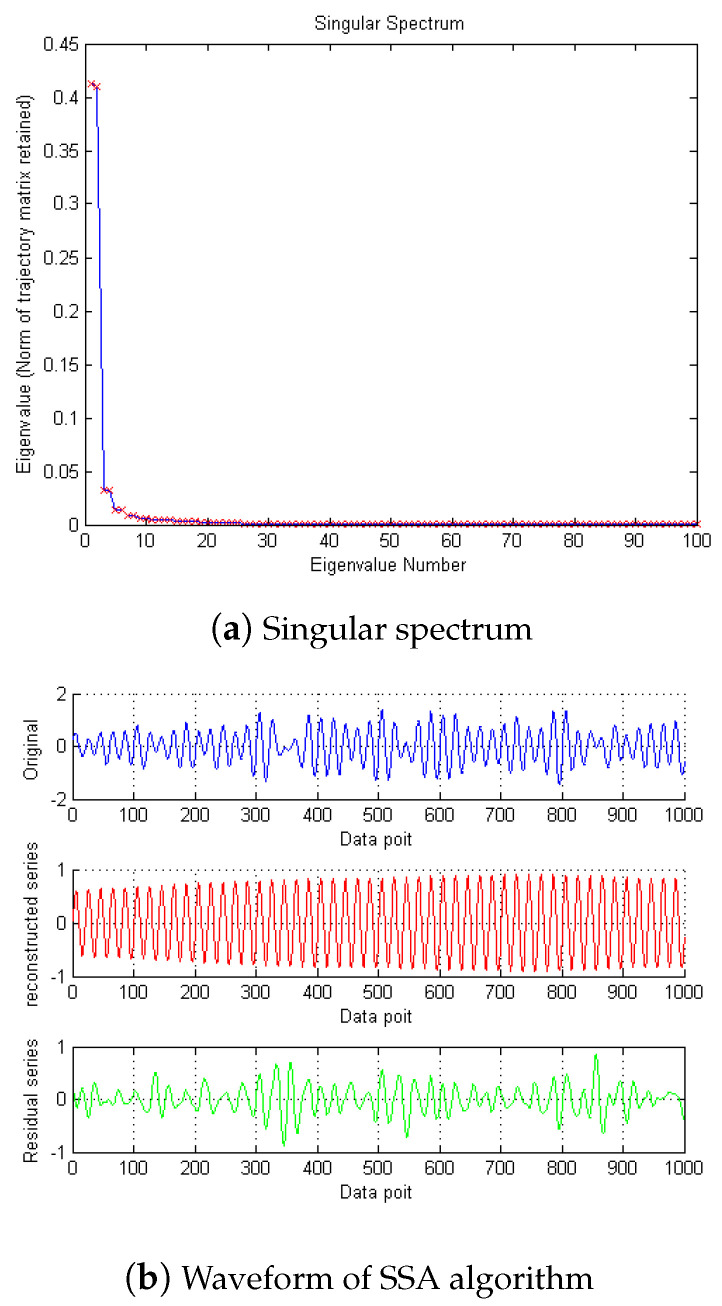
The denoising result of SSA algorithm of Equation (9) with 10 dB.

**Figure 18:**
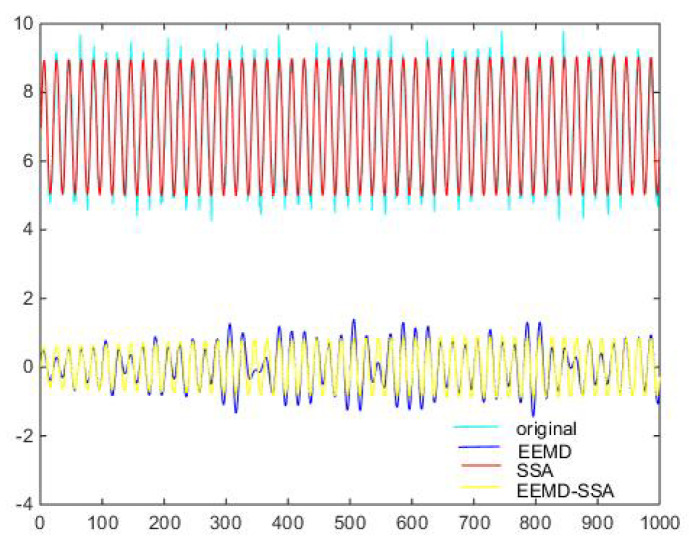
Time domain signals of different methods of Equation (9) with 10 dB.

**Figure 19:**
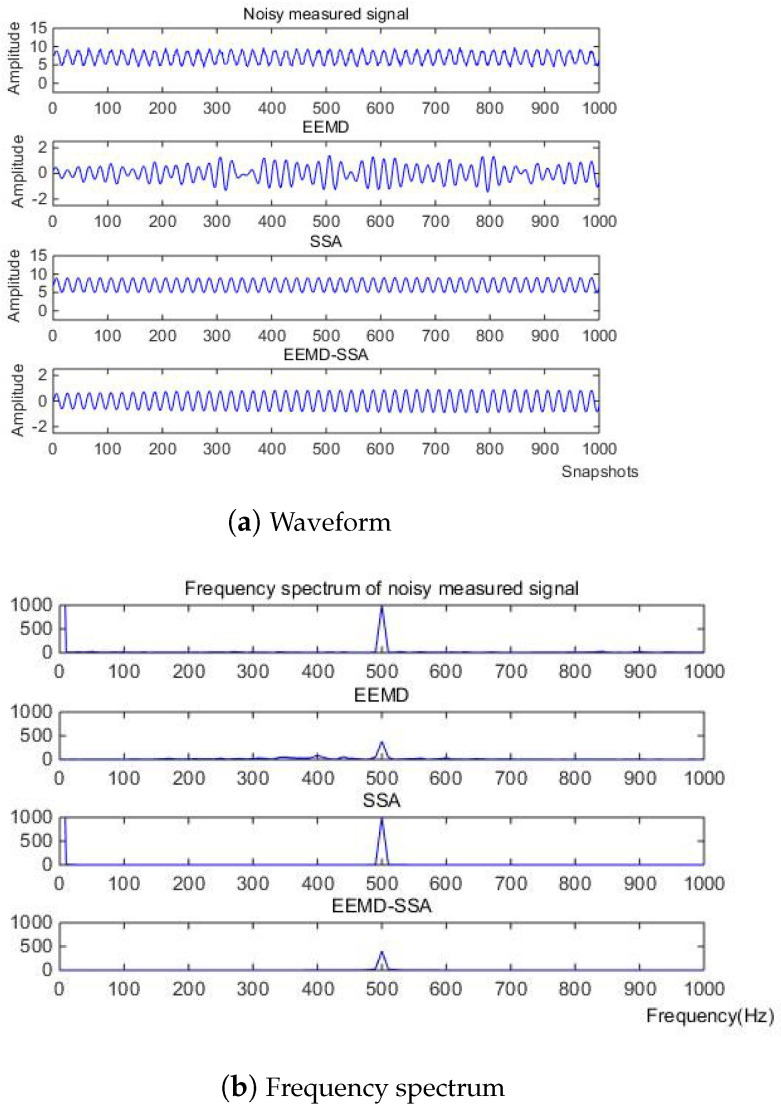
Comparison of denoising results of different algorithms of Equation (9) with 10 dB.

First, the measured noise signal is decomposed by EEMD. The time–frequency diagram is shown in Figure 15. In Figure 15, $C_{0}$ is the original signal; $C_{1}$–$C_{9}$ is ${\mathrm{IMF}}_{1}$–${\mathrm{IMF}}_{9}$; $C_{1}$, $C_{2}$, and $C_{3}$ have the lowest energy; and the frequency after $C_{6}$ is very small compared to the original signal and can be ignored, which has no effect on the result. Therefore, $C_{4}$ and $C_{5}$ are selected to be reconstructed, and the spectrum of the reconstructed signal is shown in Figure 16. Then, SSA is used for noise reduction to obtain the characteristic values of SSA and the final filtered signal and spectrum, as shown in Figure 17. According to the eigenvalue, the first singular is 0.3911, the second is 0.3882, and the third is 0.0445. Compared to the previous one, the eigenvalue after the second one is too small to be ignored; the corresponding singular value after the second singular value is the environmental noise. After removing these singular values, the denoising effect of the MEMS hydrophone is realized. In Figure 18 and Figure 19, it can be seen from the time domain and frequency domain graphs that the filtering effect is obvious.

Similarly, when mixed with the 10 dB noise, the EEMD algorithm handles the noise signal, and the baseline drift phenomenon is corrected; however, as seen in the spectrum, there is a lot of burr noise near 500 Hz. By selecting the first and second characteristic value components to reconstruct the signal and filtering out the excess noise, stable wave smoothness is achieved, and the denoising effect is great.

In order to more clearly evaluate the denoising effects of the EEMD, SSA, and EEMD-SSA methods, we conducted statistical experiments under different SNRs (−10 dB, −5 dB, 0 dB, 5 dB, and 10 dB). The experimental results are the average of 100 Monte Carlo experiments and are shown in Table 1. It can be clearly seen that EEMD-SSA can always maintain a higher signal-to-noise ratio in different situations, and its noise suppression effect is the most obvious.

## 4. Experiment and Results

**Figure 20:**
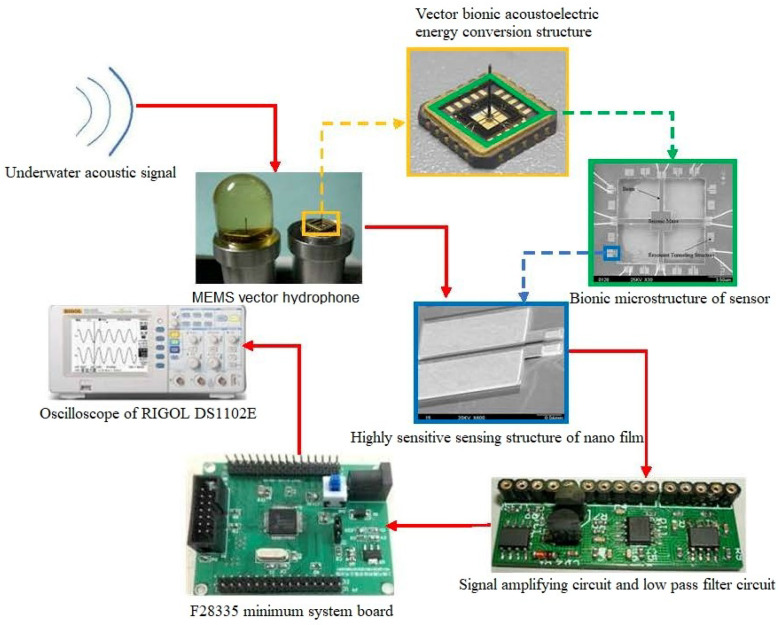
Experimental process.

**Figure 21:**
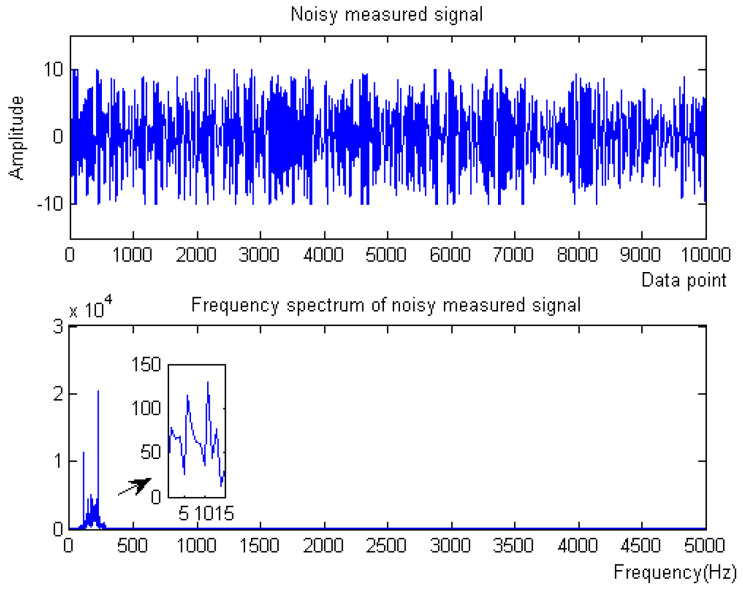
Measured signal of MEMS hydorphone.

A lake test was carried out in a reservoir in Taiyuan. A transducer was used as a sound source that emits a continuous signal with 220 Hz. The distance between the sound source and the hydrophone was about 100 m. The transducer and hydrophone were placed at the same height on the surface of the water. The process of the experiment is shown in Figure 20. The underwater acoustic signal was collected by a MEMS vector hydrophone. Through the bionic acoustic electric energy conversion structure, the acoustic signal is first converted into pressure, which deforms the highly sensitive nanofilm induction structure, converting it into an electrical signal. After an amplifying circuit and s 2 kHz low-pass filter, the collected electrical signal can be stabilized and the useless signal above 2 kHz can be filtered. Finally, the electrical signal is output to the oscilloscope after the digital filter of the F28335 minimum system board with the algorithm and a sampling rate set to 10 kHz. As can be seen from the time domain waveforms and spectrograms in Figure 21, there are still many noise components in the signal after only 2 kHz low-pass circuit due to the complexity and instability of the external field environment.

**Figure 22:**
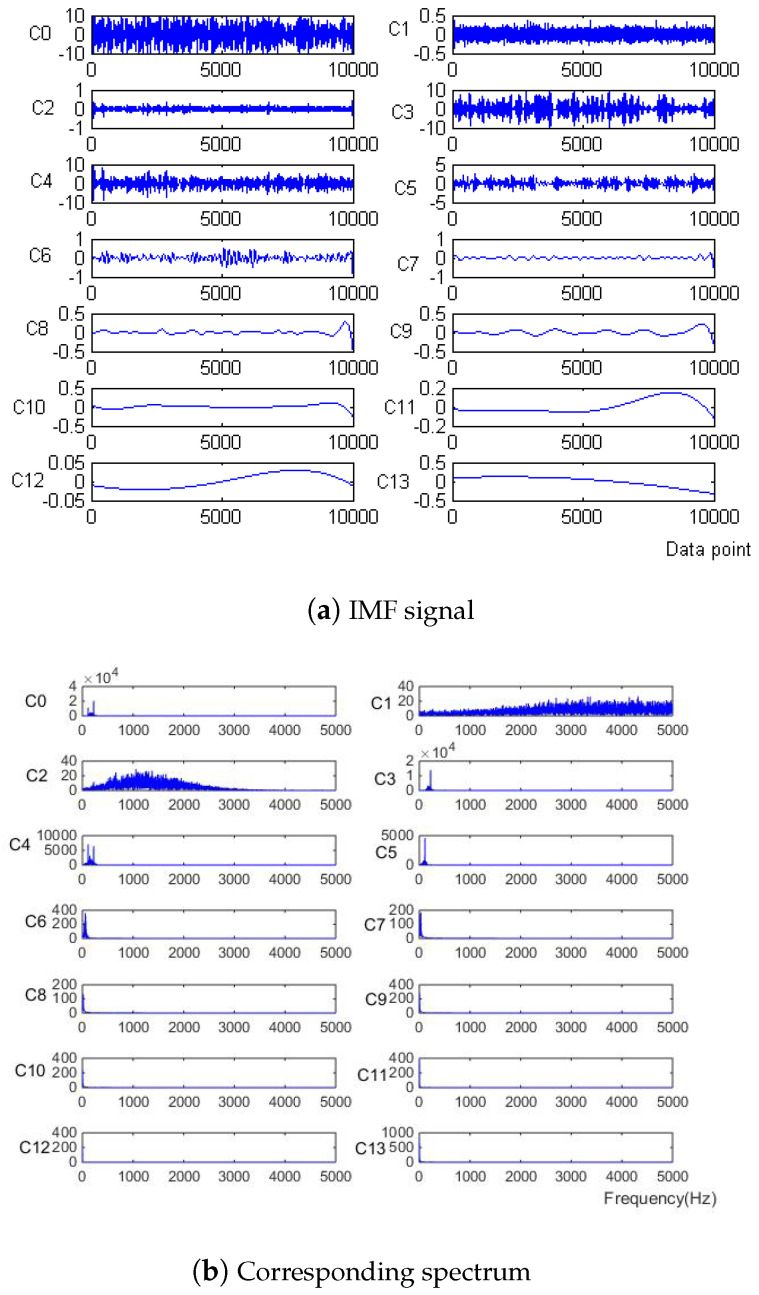
IMF signal and corresponding spectrum of measured signal.

**Figure 23:**
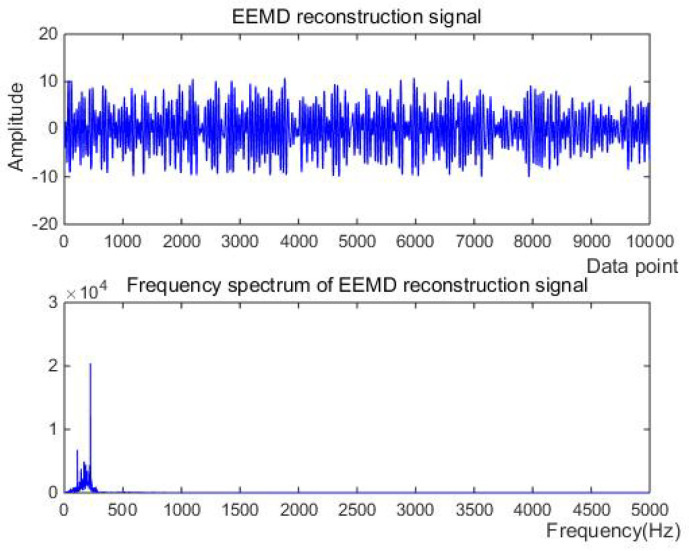
The denoising result of EEMD algorithm of measured signal.

First, the signal is subjected to EEMD, and the decomposed IMF signal and the corresponding spectrum (shown in Figure 22) have correlation coefficients of $C_{5}$–$C_{13}$ that are significantly lower than those of other IMF components. In Figure 21, $C_{0}$ is the original signal, $C_{1}$–$C_{13}$ are ${\mathrm{IMF}}_{1}$–${\mathrm{IMF}}_{13}$, and the energies of $C_{1}$ and $C_{2}$ are the lowest, so $C_{1}$, $C_{2}$, $C_{5}$–$C_{13}$ are removed and $C_{3}$ and $C_{4}$ are reconstructed. The reconstructed signal and spectrum are shown in Figure 23.

**Figure 24:**
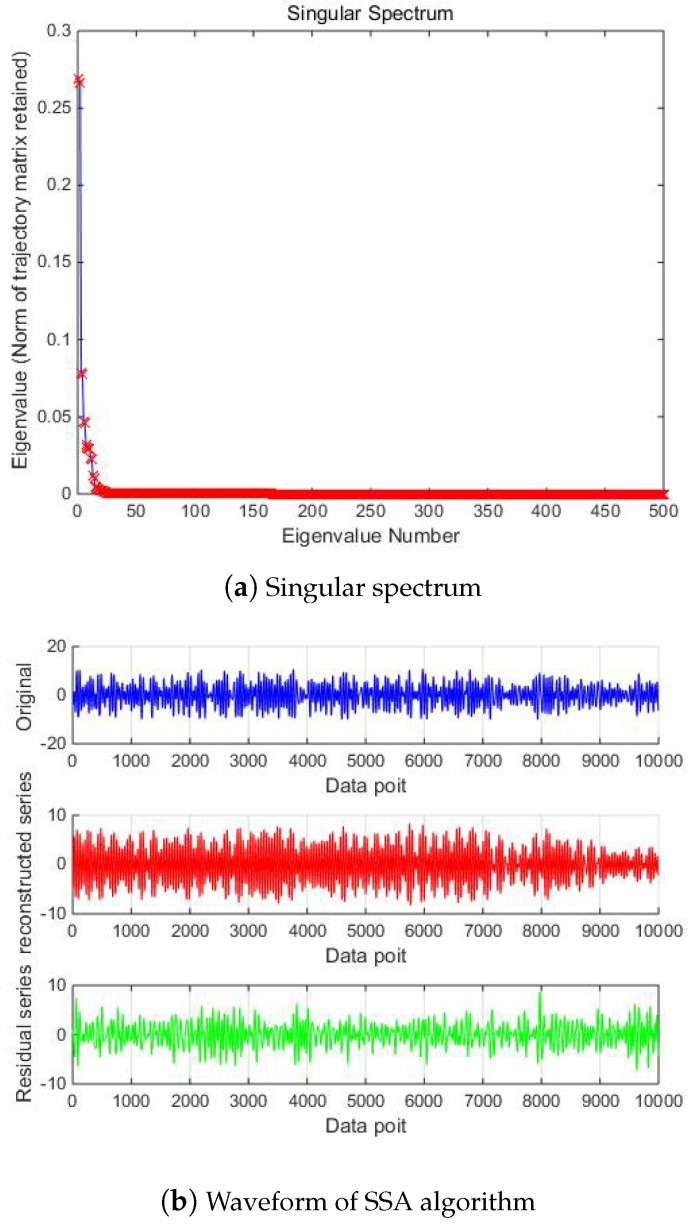
The denoising result of SSA algorithm of measured signal.

Then, SSA noise reduction is performed, and the eigenvalue graph of the SSA and the final filtered signal and spectrum are as shown in Figure 24. It can be determined from the eigenvalue graph that where the fourth singular value is 0.0773, r=4, $\lambda_{4}=0.0773$, the singular satisfies the formula

$$\frac{\frac{\lambda_{r-1}-\lambda r}{r-(r-1)}}{\frac{\lambda_{r}-\lambda_{d}}{d-r}}>1,$$

d>1, and r<d, that is, the corresponding values after the fourth singular value are environmental noise. After removing these singular values, denoising of the MEMS hydrophone is realized, and the filtering effect is obvious from the time–frequency domain diagram.

**Figure 25:**
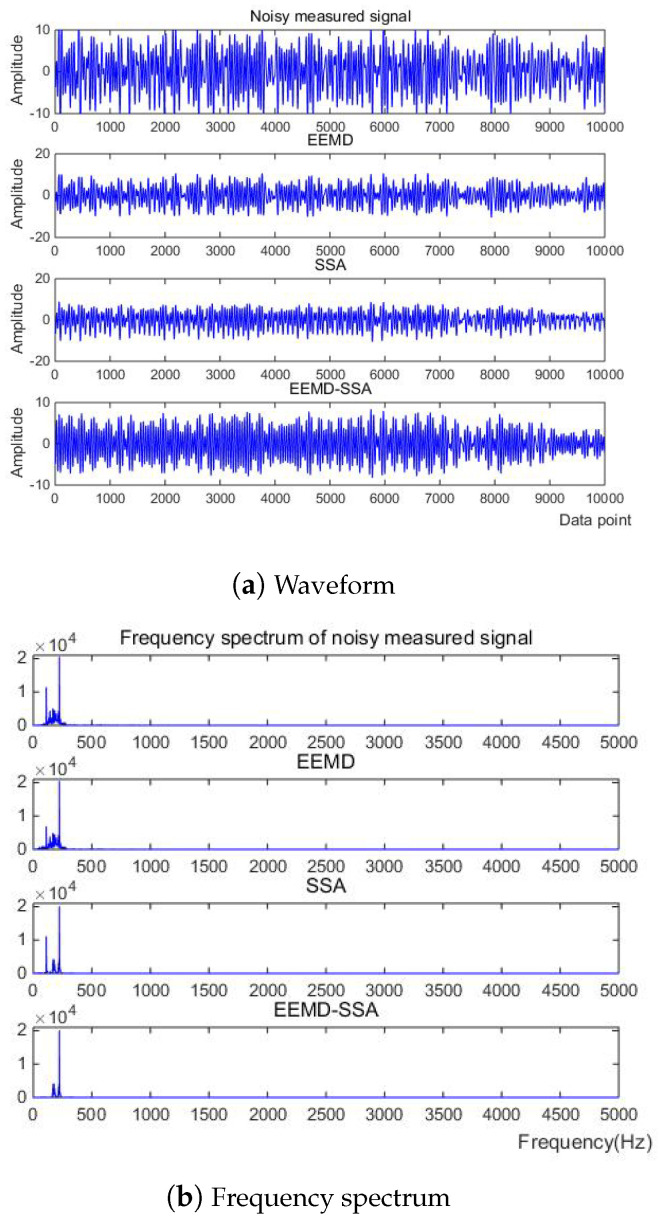
Comparison of denoising results of different algorithms of measured signal.

Then, the three methods of EEMD, SSA, EEMD-SSA are compared. The time–frequency domain diagrams are shown in Figure 25. It can be seen that the EEMD algorithm corrects the baseline drift obviously but does not improve the burr noise. After filtering using the SSA algorithm, the amplitude is stable and the waveform is smooth, but the baseline drift still exists. To summarize, the noise reduction effects of EEMD and SSA are not better than that of EEMD-SSA. Therefore, the feasibility of the method is verified.

## 5. Conclusions

In order to improve the utilization of received signals, a noise reduction technology for MEMS vector hydrophone based on EEMD and SSA is studied. The EEMD algorithm cannot completely remove noise. SSA is a method that separates the characteristic quantities from the noise signal and utilizes these quantities to reconstruct the signal for the purpose of noise reduction. Combining these two methods can effectively remove residual noise. The experimental results show that the proposed method can effectively eliminate the effects of high-frequency noise and low-frequency vibration of received signals. Note that the vibration noise is usually the maximum interference of the MEMS vector hydrophone. This method has certain guiding significance for the practical application of the MEMS vector hydrophone.

## Figures and Tables

**Table 1:** Comparison of SNR before and after denoising with different methods.

SNR	EEMD	SSA	EEMD-SSA
−10 dB	−10.8098	−2.8211	−0.0420
−5 dB	−5.9513	6.0827	7.2656
0 dB	−0.9591	13.7797	14.7295
5 dB	3.8421	17.6929	18.3387
10 dB	8.6353	22.1839	22.4746

## Data Availability

The original contributions presented in the study are included in the article, further inquiries can be directed to the corresponding author.
